# Acute Poisoning among Children Admitted in a Tertiary Care Hospital: A Descriptive Cross-sectional Study

**DOI:** 10.31729/jnma.8482

**Published:** 2024-03-31

**Authors:** Surabhi Aryal, Susmin Karki, Machhindra Lamichhane

**Affiliations:** 1 Department of Paediatrics, Tribhuvan University Teaching Hospital, Maharajgunj, Kathmandu, Nepal; 2 Maharajgunj Medical Campus, Maharajgunj, Kathmandu, Nepal

**Keywords:** *acute poisoning*, *children*, *organophosphorus*, *prevalence*

## Abstract

**Introduction:**

Acute poisoning is one of the critical causes of hospital admission in children worldwide. Understanding the clinico-demographic profile of childhood poisoning will help in developing targeted prevention strategies. This study aimed to find the prevalence of acute poisoning cases among children admitted to a tertiary care hospital.

**Methods:**

A descriptive cross-sectional study was done among 4972 children admitted in the pediatric ward, High Dependency Care Unit, and Pediatric Intensive Care Unit of a tertiary care hospital in Nepal. The data were collected from the hospital records from over three years between 1 January 2020 and 31 December 2022 after receiving ethical approval from the Institutional Review Committee. A convenience sampling method was used. Data related to the clinical and demographic data were collected from the patients with acute poisoning and analyzed. Point estimate at 95% Confidence Interval was calculated.

**Results:**

Out of 4972 paediatric cases admitted to the hospital, acute poisoning was seen in 57 (1.14%) (0.81-1.39, 95% Confidence Interval) patients. Out of these acute poisoning cases, 31 (54.39%) were accidental. The mean age was 10.10±5.40 years with 35 (61.40%) patients from the adolescent age group.

**Conclusions:**

This study conducted in a Nepalese tertiary care hospital identifies acute poisoning as a notable concern among pediatric admissions.

## INTRODUCTION

Acute childhood poisoning is a common pediatric emergency that affects millions of children globally. According to the WHO, among all the mortality due to acute poisoning, 13% occurred in age groups less than 20 years.^1^ Unintentional poisoning is common in children under five years, whereas intentional poisoning is more common in adolescents.^2-4^ Over the years, pediatric poisoning cases are rising.^5,6^ Studies from Nepal have reported prescription medication and organophosphorus (OP) poisoning as the most common agents.^7,8^

Although most cases of childhood poisoning are non-lethal, the consequences of some childhood poisoning extend far beyond immediate harm with long-term health complications.^9^ The clinical severity depends upon various factors and more studies are required to recognize poisoning trends for developing targeted prevention strategies, improving medical interventions, and reducing the incidence and severity.

This study aimed to find the prevalence of acute poisoning cases among children admitted to a tertiary care hospital.

## METHODS

This descriptive cross-sectional study was carried out in 4972 children admitted in the pediatric ward, High Dependency Care Unit, and Pediatric Intensive Care Unit of a teaching hospital in Kathmandu during the period of three years from 1 January 2020 to 31 December 2022. The Institutional Review Committee of the institute approved ethical clearance (Reference Number: 436(6-11)E2 079/080). Children aged 16 years and younger who were admitted to the hospital were included in the study. Children with food poisoning and records with incomplete data were excluded from the study. The sample size was calculated using the following formula:


$$\begin{array}{ll} \text{n} & ={\text{Z}}^{2}\times \frac{\text{p}\times \text{q}}{{\text{e}}^{\text{2}}}\\ & =1.96^{2}\times \frac{0.50\times 0.50}{0.02^{2}}\\ & =2402 \end{array}$$

Where,

n = minimum required sample size
Z = 1.96 at 95% Confidence Interval (CI)
p = prevalence taken as 50% for maximum sample size
q = 1-p
e = margin of error, 2%

The minimum required sample size required was 2402. A final sample of 4972 children was taken. A convenience sampling method was used for sample collection. Acute poisoning cases were identified with the definition of a brief exposure (less than 24 hours) to a toxic substance.^10^ The main outcome was the prevalence of acute poisoning cases among the admitted children. Data regarding age, sex, type of poisoning agent, place of poisoning, presenting symptoms and signs, duration of hospital stay, and outcome were collected from the hospital records. A checklist for data collection, comprising the variables selected for measurement, was made and used for gathering the required data. Upon collection, the data underwent thorough checks for completeness, accuracy, and consistency. Subsequently, they were organized and securely stored in preparation for compilation and analysis. Descriptive statistics was used to determine mean and standard deviations for the continuous variables and absolute numbers and percentages for the categorical variables. Point estimate at 95% CI was calculated.

## RESULTS

Out of 4972 cases admitted to the hospital, acute poisoning was seen in 57 (1.14%) (0.81-1.39, 95 % CI) patients. The age of the children ranged from 11 months to 16 years, with the mean age being 10.10±5.40 years. A total of 35 (61.40%) children with acute poisoning belonged to the adolescent age group (10-16 years), followed by the age group less than five years 19 (33.33%). There were 37 (64.91%) females with an female: male ratio of 2.8:1, but in age groups less than five years and six years to ten years, the female: male ratio was 1.1:1 and 2:1, respectively.

Accidental poisoning was seen in 31 (54.38%) cases, of which 19 (61.29%) were under five. OP compound was used in 11 (47.82%) of suicidal poisoning. In 49 (85.96%) cases, the event happened at home. Most of the poisoning was between 6 pm to midnight in 19 (33.33%) cases, followed by 12 pm to 6 pm in 17 (29.82%) cases. The route of poisoning was ingestion in 49 (85.96%), bite/sting in 6 (10.52%), inhalation in 1 (1.75%), and contact in 1 (1.75%) (Table 1).

**Table 1:** Gender, mode, and types of poison according to age group (n = 57).

	<5 years n (%)	5-10 years n (%)	>10 years n (%)	Total n (%)
**Sex**				
Male (n = 20)	9 (45.00)	1 (5.00)	10 (50.00)	20 (35.09)
Female (n = 37)	10 (27.04)	2 (5.40)	25 (67.56)	37 (64.91)
**Mode**				
Accidental (n = 31)	19 (61.29)	2 (6.45)	10 (32.25)	31 (54.40)
Suicidal (n = 23)	-	-	23 (100)	23 (40.35)
Curiosity (n = 1)	-	-	1 (100)	1 (1.75)
Unknown (n = 2)	-	1 (50.00)	1 (50.00)	2 (3.50)
**Type of poison**				
Organophosphate (n = 15)	-	2 (13.33)	13 (86.66)	15 (26.32)
Drugs (n = 16)	8 (50.00)	1 (6.25)	7 (43.75)	16 (28.00)
Rodenticides (n = 5)	-	-	5 (100.00)	5 (8.77)
Corrosives (n = 5)	5 (100.00)	-	-	5 (8.77)
Herbicide (n = 3)	1 (33.33)	-	2 (66.66)	3 (5.26)
Dhatura (n = 2)	1 (50.00)	-	1 (50.00)	2 (3.50)
Envenomation (n = 6)	2 (33.33)	-	4 (66.66)	6 (10.52)
Carbon monoxide (n = 1)	-	-	1 (100.00)	1 (1.75)
Insect repellent (n = 4)	1 (25.00)	-	3 (75)	4 (7.01)

Out of 16 children with acute poisoning who took drugs, subclassification was done based on the different types of drugs they took (Table 2).

**Table 2:** Types of drugs taken by the patients with acute poisoning (n = 16).

Drug	n (%)
Paracetamol	5 (31.25)
Anti psychotics	5 (31.25)
Levetiracetam	1 (6.25)
Salbutamol	1 (6.25)
Thyroxine	1 (6.25)
Anti depressant	1 (6.25)
Antibiotic (Amoxicillin)	1 (6.25)
Benzodiazepine	1 (6.25)

The earliest presentation time after the event was 45 minutes for a case of paracetamol poisoning, and the latest was seven days for a case of snake bite, which was referred from another center for intensive care. Out of the total, 17 (29.82%) cases reached our center within 2 hours, and 30 (52.63%) cases reached our center within 2-12 hours. A total of 7 (12.28%) children arrived 24 hours after the event, of which 6 (10.52%) were referred from another center after initial management.

Similarly, 51 (89.47%) of cases were symptomatic at presentation, with vomiting being the most common symptom 32 (56.14%), followed by decreased consciousness 15 (26.32%). Tachycardia was seen in 18 (31.58%). A total of 7 (12.28%) had both tachycardia with tachypnea. Fifteen patients (26.32%) had Glasgow Coma Scale <15 at presentation, of which 6 (40%) were poisoned by drugs, and 5 (33.33%) were Organophosphate (OP) poisoning.

Four cases (7.02%) had received first aid at home, and 27 (47.37%) patients had received some form of treatment in a healthcare facility before the presentation. Twelve (85.71%) out of fourteen cases of OP poisoning received an antidote. The maximum and minimum duration of hospital stay was 35 days (case of snake envenomation) and 16 hours (Amoxicillin overdose), respectively, with a median of 3 days. Eighteen (31.58%) patients were admitted for only one day. Out of the total cases, 38 (66.67%) cases required hospitalization for more than seven days. Sixteen cases (28.07%) received care in the Pediatric Intensive Care Unit (PICU), and four required mechanical ventilation. One (6.67%) case out of 15 OP poisoning cases required a mechanical ventilator. One (1.75%) case of OP poisoning left against medical advice on the fifth day. Among the total cases, 2 (3.51%) cases of herbicide (paraquat) poisoning and 1 (1.75%) case of wasp bite required hemodialysis. One (1.75%) mortality case of herbicide (paraquat) poisoning expired on the 14^th^ day of paraquat ingestion.

## DISCUSSION

The study aimed to determine the prevalence of acute poisoning cases among children aged ≤16 years admitted to a tertiary care hospital. The key results revealed that out of the 4972 cases admitted, acute poisoning was observed in 57 (1.14%) of patients, with 31 (54.39%) of cases being accidental. The mean age was 10.10±5.40 years, and the adolescent age group (10-16 years) represented the majority at 35 (61.40%).

The real incidence of childhood poisoning outnumbers the available data in Nepal, as most cases are unreported. Children of all age groups are at risk of poisoning, with two peaks of age in children under five years because of curiosity and constantly exploring nature and lack of awareness of potential risk and again during adolescence, possibly because of intentional self-harm behavior. Most studies have reported that poisoning is commonest in children under five years.^4,7,11^ However, in this study, the highest number of poisoning was seen in the age group of more than ten years, which is consistent with the study done by Lin et al., and the majority of which was suicidal.^12^ This finding may be explained by the increasing rate of intentional poisoning among adolescents in Nepal. Females were more than males, similar to a study by Bacha et al.^13^ However, the difference was less in the age group <5 years. This finding is similar to a study in rural Sri Lanka,^14^ where females significantly predominated in older children. This could suggest a potential regional influence on gender distribution in childhood poisoning.

In the majority of cases, the event occurred at home, which is similar to other studies.^3,5,7^ The reason may be that household chemicals such as pesticides, cleaning products, or medications are usually stored at home within easy reach of children and without proper labeling. The type of poisoning agents depends on geographic location, as reported in different studies, with pesticides common in rural areas and pharmaceutical agents in urban areas. Moreover, the common poisoning agent has also changed over time, with a decrease in kerosene poisoning cases owing to the use of alternative fuel sources in recent days.^15^ Most children in this study were poisoned by pharmaceutical medications followed by organophosphorus compounds. Nepal is an agricultural country, and organophosphorus compounds are easily available and accessible at home. Medications were the most common agent for poisoning in studies done by Dhakal et al.^7^ in Nepal, Lee et al.^11^ in Taiwan, and Shirkosh et al.^5^ in Iran. This data highlights the importance of storing medications in child-resistant containers and places not easily accessible to children. Paracetamol, the antipyretic that is easily available over the counter, is an essential agent in childhood poisoning, as observed in this study. The availability of Paracetamol in different dose concentrations aids in accidental paracetamol poisoning. Among household products, cleaning agents and pesticides were the most common poisoning agents. This is a similar finding to previous studies done in Malaysia^16^ and the United States,^17^ which also reported cleaning liquids as the common household product in childhood poisoning. Studies from Nepal have reported prescription medication and OP poisoning as the most common agents.^7,8^

In our study, the majority of the cases were symptomatic at presentation in contrast to other similar studies. Primary symptoms depend on the type of poison. In our study, vomiting was the most common symptom, followed by neurological symptoms, explained by the high frequency of neurotoxic agents. Most of the cases arrived after two hours of the event, possibly because almost half of the patients were referred after initial management in other centers and due to difficulty accessing health facilities.

The duration of hospital stay depends on the type and quantity of poison ingested and the severity of symptoms. The mean hospital stay time in various studies ranges from 0.66^16,18^ to 17 days.^11,13^ The mean time of stay in this study was 5.3 days. In our study, most organophosphorus compounds required hospitalization for more than seven days, out of which only one required a mechanical ventilator (6.66%), and all survived. A study by Baseer et al. in Egypt mentioned that 13% of cases of OP poisoning required a mechanical ventilator with 5% mortality.^19^ The better outcome of OP poisoning in our study may be due to prompt resuscitation and initiation of antidotes. Various studies have mentioned no death to 3.4% mortality in childhood poisoning.^3,11,20^ The case fatality rate in our study was 1.75%. Low mortality might be due to the quantity of poison that children ingest is usually below the lethal range. Additionally, there is an overall decrease in childhood poisoning-related deaths worldwide due to improved emergency and intensive care.^20^

The variations observed in the results might stem from contextual factors such as regional differences in poison availability, socio-cultural norms influencing healthcare-seeking behavior, and diverse healthcare infrastructure. Additionally, differences in study designs, definitions, and data collection methods across studies may contribute to discrepancies in reported prevalence rates. Clinically, our findings emphasize the importance of healthcare professionals being vigilant about the types of poisoning agents prevalent in the region. This knowledge can guide prompt and effective interventions, particularly in cases of pharmaceutical medication poisoning, where timely administration of antidotes is critical.

Several limitations must be acknowledged. Being a retrospective, single-center study, the generalizability of our findings to the broader population is limited. The reliance on hospital admissions may underestimate the true incidence of childhood poisoning, as milder cases may be managed outside the hospital setting. Additionally, the accuracy and completeness of recorded data in medical records introduce potential sources of bias and imprecision.

## CONCLUSIONS

This study conducted identifies acute poisoning as a notable concern among pediatric admissions in a Nepalese tertiary care hospital. Accidental poisoning is predominant, challenging the perception of higher risk in younger children. Adolescents, particularly females, are disproportionately affected. Home environments emerge as key locations for incidents, emphasizing the need for enhanced safety measures. Pharmaceuticals and organophosphorus compounds are the leading agents. The study highlights the necessity for targeted preventive strategies and increased awareness to address the specific challenges of childhood poisoning in the Nepalese setting.
